# Benthic Feeding and Diet Partitioning in Red Sea Mesopelagic Fish Resolved Through DNA Metabarcoding and ROV Footage

**DOI:** 10.1002/ece3.71091

**Published:** 2025-03-06

**Authors:** Kah Kheng Lim, Carlos Angulo‐Preckler, Christopher A. Hempel, Mohammad A. Qurban, Vincent A. Pieribone, Carlos M. Duarte

**Affiliations:** ^1^ Marine Science Program, Biological and Environmental Science and Engineering Division King Abdullah University of Science and Technology (KAUST) Thuwal Kingdom of Saudi Arabia; ^2^ National Center for Wildlife (NCW) Riyadh Kingdom of Saudi Arabia; ^3^ OceanX New York New York USA

**Keywords:** biological carbon pump, myctophids, phosichthyids, trophic ecology, western Indian Ocean

## Abstract

Mesopelagic fish are among the most abundant vertebrates on Earth and play a crucial role in carbon sequestration through their daily vertical migration. However, their dietary ecology remains poorly understood, especially in the Red Sea, limiting our grasp of their trophic interactions and ecological roles. This study investigates the dietary composition of two common mesopelagic fish species in the Red Sea, the lanternfish (*Benthosema* taxa) and the endemic lightfish ( *Vinciguerria mabahiss* ), using DNA metabarcoding of the mitochondrial COI marker, supplemented by remotely operated vehicle (ROV) video observations. Our findings show that *V. mabahiss* exhibits higher prey diversity compared to *Benthosema* taxa, suggesting a more generalist feeding strategy. Both species primarily consume copepods, likely due to the high abundance of copepods in the upper 200 m of the Red Sea. Despite this commonality, distinct dietary niches were observed: *Benthosema* taxa consumes significant amounts of molluscs, followed by annelids and echinoderms, while *V. mabahiss* occasionally consumes gelatinous prey such as hydrozoans and scyphozoans. Notably, our ROV video footage demonstrates that these mesopelagic fish engage in benthic feeding on the continental slope, a behavior rarely documented. By consuming and redistributing organic material through their diel vertical migrations, mesopelagic fish contribute to the biological carbon pump, with important implications for carbon sequestration processes in the ocean. Future studies integrating DNA metabarcoding with stable isotope analysis could provide deeper insights into dietary partitioning and the ecological contributions of these mesopelagic fish species to the Red Sea ecosystem and beyond.

## Graphical abstract

Our study combined DNA metabarcoding and ROV footage to reveal that mesopelagic fish in the Red Sea engage in benthic feeding, a behavior previously unreported for these species. This finding suggests an additional carbon sequestration pathway, highlighting the need to reassess their role in carbon cycling within the mesopelagic ecosystem.

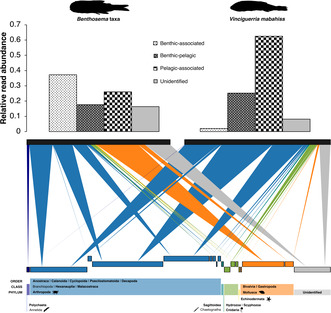

## 1 Introduction

The mesopelagic layer, extending from 200 to 1000 m, is a fundamental component of the ocean ecosystem linking the biogeochemical and ecosystem dynamics of the overlying upper ocean and the underlying deep sea (Davison et al. 2013). Mesopelagic fish, supporting the largest fish stock in the world ocean with a recent estimate of approximately 10 billion tonnes (Irigoien et al. 2014), play a pivotal role as both predators and prey in intricate food webs (Cherel et al. 2010). Mesopelagic fish perform diel vertical migration (DVM; Klevjer et al. 2016), which profoundly impacts carbon and nutrient cycling and facilitates energy transfer between surface and deeper layers, thereby playing a critical role in biogeochemical cycles, including the active ocean biological carbon pump (Hernández‐León et al. 2020; Irigoien et al. 2014; Robinson et al. 2010).

The Red Sea presents a unique environment for studying mesopelagic fish behavior and ecology due to its dense and distinct deep‐scattering layers primarily comprised of fish (Kaartvedt et al. 2024, 2019; Klevjer et al. 2012). Among the most abundant species are the lanternfish *Benthosema pterotum* and the lightfish *Vinciguerria mabahiss* , whose larvae dominate the mesopelagic fish community (Abu El‐Regal and Ditty 2023; Isari et al. 2017), underscoring their significance in the ecosystem's trophic dynamics and carbon cycling (Cherel et al. 2010). The high density and abundance of these species, contrasted with the oligotrophic nature of the Red Sea (Qurban et al. 2014; Raitsos et al. 2013), support the hypothesis that efficient carbon transfer processes are essential for sustaining the biomass of mesopelagic fish (Irigoien et al. 2014). Thus, understanding how mesopelagic fish meet their dietary requirements necessitates a detailed knowledge of their prey items.

Research has shown a high variation in the biodiversity and size of prey items consumed by lanternfish species, which may be attributed to their life stages, behavior, and anatomical features (Dalpadado and Gjosaeter 1988; Dypvik and Kaartvedt 2013; Loutrage et al. 2024; Martin and Davis 2020). While mesopelagic fish are generally understood to have a crustacean‐dominated diet (Dalpadado and Gjosaeter 1988; Dypvik and Kaartvedt 2013), detailed comparative studies, particularly in oligotrophic environments like the Red Sea, are lacking. Previous studies have indicated that *B. pterotum* and *V. mabahiss* occupy overlapping ecological niches within the deep‐scattering layer, with some degree of vertical overlap and mixing between their depth ranges (Dypvik and Kaartvedt 2013; Kaartvedt et al. 2024). However, a detailed understanding of the dietary habits of these species, particularly in interspecies comparisons, remains limited.

This study addresses these gaps by integrating DNA metabarcoding and remotely operated vehicle (ROV) footage to elucidate the dietary preferences and trophic interactions of *Benthosema* taxa and *V*. *mahabiss*. Utilizing Cytochrome Oxidase 1 (COI) primer sets through next‐generation sequencing (NGS), this study overcomes traditional sampling limitations, providing a comprehensive analysis of gut contents, including soft‐bodied and highly digested prey (Leray et al. 2015, 2013). This approach reveals detailed insights into prey composition and diet partitioning but also offers potential detection of cannibalism and intra‐specific interactions, although some limitations exist due to primer affinity with predator DNA and the need for high sequencing depth. The integration of ROV footage further highlights benthic feeding behaviors—a phenomenon rarely documented in mesopelagic fish—which may reflect an adaptive response to resource availability in oligotrophic environments. By linking these findings to broader ecological processes, such as food web dynamics and energy flow between pelagic and benthic systems, our study advances understanding of the ecological roles and evolutionary strategies of mesopelagic fish in shaping trophic interactions and ecosystem functioning in the Red Sea and similar environments.

## 2 Materials and Methods

### 2.1 Fish Collection and ROV Video Analysis

**FIGURE 1:**
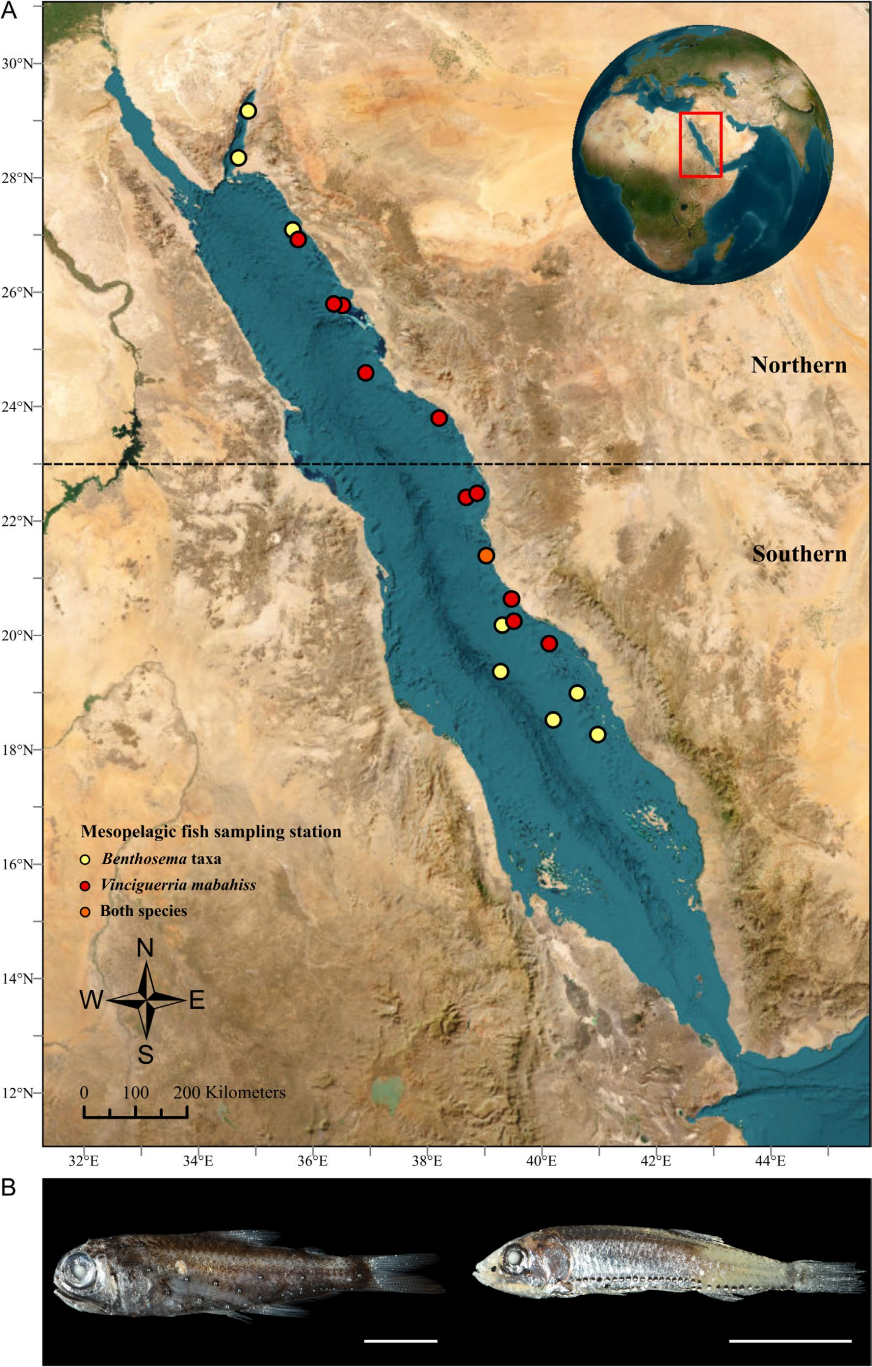
(A) Sampling locations of mesopelagic fish along the Saudi Arabian Red Sea during the Red Sea Decade Expedition; (B) High‐resolution images of *Benthosema* taxa (left) and *Vinciguerria mabahiss* (right). Orange dot indicates the sampling station where both species were captured. Scale bars represent 10 mm.

All fish were collected as bycatch of animals attracted to the lights of the ROVs and impacted by the motion of the thrusters during ROV activities conducted as part of the Red Sea Decade Expedition, conducted between February and June 2022. Sampling was carried out at 19 stations along the continental slope of the Red Sea, at depths of 252–818 m, corresponding to the mesopelagic scattering layer (200–800 m) and the migration profile of mesopelagic fish in the region (see Kaartvedt et al. 2024 and references therein). Most sampling (*n* = 17) occurred during the day (between 06:00 and 12:00) near the seafloor when ROV intersects their downward migration. One‐minute ROV video clips (*n* = 17) were analyzed to document mesopelagic fish behavior, focusing on their interactions and potential feeding within the benthic environment. Behaviors such as swift darting near the seafloor and predation by benthic‐dwelling organisms were noted. Sampling stations in the Red Sea were grouped into two provinces (Northern vs. Southern) based on the distribution of our samples (Figure 1, Table S1). Upon collection, the fish were stored at −20°C and transported back to King Abdullah University of Science and Technology (KAUST). At KAUST, each fish was photographed, measured, weighed, morphologically identified, and stored in 70% ethanol prior to DNA extraction (Table S2 in Data S1). The research was performed under ethics approval (23IBEC055).

### 2.2 Sample Preparation and DNA Extraction

Stomach contents were extracted from 30 individuals of *Benthosema* taxa and 30 individuals of *Vinciguerria mabahiss* using a pair of microdissection scissors (3 mm cutting edge, Khosla, Mumbai, India) and forceps under a stereomicroscope. Dissecting tools were sterilized between individuals by rinsing them first in 10% bleach, then Milli‐Q water, and finally 70% ethanol. The community DNA of the gut content of each sample was extracted using the DNeasy PowerSoil Pro kit, following the manufacturer's protocol. This kit was selected for its proven efficacy in recovering prey DNA, as demonstrated in previous dietary metabarcoding studies on small‐bodied fish (Palacios‐Narváez et al. 2024). The integrity of the extracted DNA was checked using the Qubit 1X dsDNA HS Assay kit on a Varioskan Lux multimode microplate reader (Thermo Fisher Scientific, Waltham, MA, USA).

### 2.3 DNA Amplification, Library Preparation, and Next Generation Sequencing

To study the gut communities, the mitochondrial COI region was amplified using the “mlCOIintF” and “jgHCO2198” primers (Wangensteen et al. 2018) with Illumina overhang adapters. The polymerase chain reaction (PCR) was conducted following the Universal Multiplex Cycling Protocol recommended by QIAGEN. The PCR cocktail consisted of 1× QIAGEN Multiplex PCR master mix (QIAGEN, Valencia, CA, United States), 0.2 μM of both forward and reverse primers, 3 μL of genomic DNA, and 8.5 μL of RNase‐free water, totaling 25 μL. PCR was conducted on a Applied Biosystems SimpliAmp thermal cycler (Thermo Fisher Scientific, Waltham, MA, USA). The thermal cycling profiles consisted of an initial denaturation at 95°C for 15 min, followed by 35 cycles of denaturation at 94°C for 30 s, annealing at 55°C for 90 s, extension at 72°C for 90 s, and a final elongation step at 72°C for 10 min. PCR products were visualized on a 1.5% agarose gel (1xTAE) stained with SYBR Safe (Invitrogen, Carlsbad, CA). For PCR negative controls, the DNA template was substituted with RNase‐free water. All PCR products were cleaned with AMPure XP beads using a bead‐to‐sample ratio of 0.8×, following the Illumina library preparation workflow until PCR Clean‐Up 2. The concentration of the amplification products was quantified using a Varioskan Lux multimode microplate reader (Thermo Fisher Scientific, Waltham, MA, USA) using the Qubit 1X dsDNA HS Assay kit and pooled in equimolar ratios. The pooled samples were quantified using the Qubit 4.0 fluorometer (Thermo Fisher Scientific, Waltham, MA, USA) and loaded on an Illumina Novaseq 6000 2×150 bp flow cell at 10 pM at the Bioscience Core Lab, KAUST.

### 2.4 Bioinformatic Processing

Demultiplexing was carried out by the sequencing facility. To accurately reflect species composition within the gut content community, especially for metazoans exhibiting high intraspecific polymorphism in the COI gene (Brandt et al. 2021), we initially obtained Exact Sequence Variants (ESVs) and subsequently clustered them into Operational Taxonomic Units (OTUs) as recommended by the literature (Antich et al. 2021; Brandt et al. 2021; Porter and Hajibabaei 2020). This two‐step approach was implemented to balance the high resolution of ESVs with the potential biological relevance of OTUs in our study. Specifically, paired‐end reads were processed using a modified version of Apscale v1.6.3 (Buchner et al. 2022), in which Swarm (Mahé et al. 2022) was implemented as an alternative to vsearch (Rognes et al. 2016) for OTU clustering. The modified pipeline also included DnoisE (Antich et al. 2022) as an alternative to unoise for denoising, with the option to first denoise reads before clustering the denoised reads into OTUs. Additionally, microDecon (McKnight et al. 2019) was used to remove reads found in PCR negative controls from true samples. This modified version of the Apscale pipeline was executed as follows: the maximum expected error (maxEE) was set to 2 (discarding reads with more than 2 expected errors), minimum and maximum read lengths were set to 303 bp and 323 bp, respectively (discarding merged reads not within the target amplicon length), reads were denoised using DnoisE with default parameters before being clustered into OTUs using Swarm, and default parameters were applied for paired‐end merging, primer trimming, dereplication, pooling, and LULU filtering (Frøslev et al. 2017). The modified version of Apscale is available on GitHub (https://github.com/hempelc/apscale), as well as a wrapper to run apscale in the command line (https://github.com/hempelc/apscale_wrapper).

We assigned taxonomy to the OTUs using BLAST (Altschul et al. 1990) against the MIDORI2 COI database (Leray et al. 2022) v257 (MIDORI2_UNIQ_NUC_GB257_COI_BLAST) using an *E*‐value threshold of 1e‐05. We filtered BLAST hits as follows: (1) all hits with a bitscore < 150 and an alignment length of < 100 were excluded, (2) for every OTU, all hits whose bitscore did not fall within a 2% margin of the highest bitscore were excluded, (3) if multiple taxa occurred among the remaining hits, only their Lowest Common Ancestor was retained, and (4) taxonomic lineages were trimmed based on percentage identity to the query; specifically, we trimmed at 98%, 95%, 90%, 85%, 80%, and 75% for the species, genus, family, order, class, and phylum levels, respectively, meaning that only hits with a percentage identity score of > 98% were assigned to the species level and so forth. This stringent filtering approach ensured the minimization of false‐positive detections.

### 2.5 Data Analyses and Visualization

We removed singletons, terrestrial contamination, and non‐target host sequences from the dataset (i.e., three OTUs identified as Myctophidae and Phosichthyidae) (Table S2 in Data S1). The relative abundances of the top 10 abundant phyla were visualized using bar charts. To understand the distribution of prey reads of mesopelagic fish, all OTUs were initially classified into broad taxonomic categories: fungi, protists, algae, metazoans, and unidentified sequences. Focusing on metazoans, we then employed a stepwise zooming approach, examining the most abundant taxa composition progressively at the phylum, class, and order levels.

Data analysis and visualization were conducted in R (R Core Team, 2024) using the packages: microViz (Barnett et al. 2021), phyloseq (McMurdie and Holmes 2013), ggvenn (Yan 2023), vegan (Oksanen et al. 2015), tidyverse (Wickham et al. 2019), colorRamps (Keitt 2024), and bipartite (Dormann et al. 2008). The alpha diversity of the prey items (richness, Shannon, and Simpson indices) of the two host species was calculated using the estimate_richness() function from the phyloseq package. Beta diversity between the two host species was illustrated using non‐metric multidimensional scaling (NMDS) based on the robust Aitchison distance (Martino et al. 2019). To test for similarity among species and provinces from which individuals were sampled, we performed a permutational analysis of variance (PERMANOVA) on the robust Aitchison distance matrix, using the adonis2() function in vegan, with species and province as predictor variables with 9999 permutations. Following PERMANOVA, we tested the homogeneity of group dispersions using the betadisper() function in vegan to ensure the significant results from PERMANOVA were not influenced by differences in dispersion among groups.

To explore the dietary preferences of each host species, we constructed a bipartite network representing associations between higher‐level metazoan prey taxa and the two fish species based on the relative abundances of prey OTUs. To streamline the analysis, we filtered out all OTUs with < 0.5% relative abundance across both species. Subsequently, we taxonomically classified the remaining OTUs: those identified with at least a 95% sequence identity were assigned to the order level, while those with a match between 75%–95% identity were assigned to the class level. OTUs with less than a 75% identity match were classified at the phylum level. Using this dataset, prey items were categorized as pelagic, benthic, or benthic‐pelagic (representing taxa that could inhabit both environments) based on their adult life stages, with any non‐assigned taxa labeled as unidentified. We also performed indicator species analysis to identify prey taxa that were specifically associated with one fish species over the other. This method tests for both fidelity (the probability that a prey taxon occurs within a particular fish species) and specificity (the degree to which a prey taxon is restricted to one fish species). The analysis was based on the relative abundance of each prey OTU in the gut contents, and we used this to determine which prey taxa were indicators of the diet for each host species.

## 3 Results

### 3.1 Gut Prey Communities

A total of 120,074,724 reads, binned into 826 Operational Taxonomic Units (OTUs), were retrieved from 60 gut samples of the two studied species. Host reads assigned to *Benthosema* taxa and *Vinciguerria mabahiss* constituted 89.4% of the total reads and were omitted from further analysis. The remaining reads (10.6%) consisted of fungi, protists, algae, metazoans, and unidentified sequences. Algae, predominantly Chlorophyta, constituted 70% of the total prey reads. Metazoans accounted for 25% of the reads, while unidentified sequences, fungi, and protists accounted for 3%, 1%, and 0.6% of the reads, respectively. Within the metazoans, arthropods were the most abundant taxon, with copepods (referred to as class Hexanauplia in our dataset due to former classification) as the dominant group. Calanoid copepods were the most abundant arthropods, followed by poecilostomatoid and cyclopoid copepods. Additionally, both fish species consumed chaetognaths, molluscs, and cnidarians, reflecting a broad dietary spectrum.

Our analysis revealed that apart from arthropods, the diet of mesopelagic fish included a significant portion of benthic prey. *Benthosema* taxa notably consumed hard‐bodied prey such as annelids, molluscs, and echinoderms, which are indicative of benthic interactions. Although *V. mabahiss* showed a lesser degree of benthic prey consumption, it still demonstrated a mixed diet of planktonic and benthic organisms. This mixed diet reflects the unique ecological setting of the continental slope, where mesopelagic fish capitalize on resources from both planktonic and benthic communities. This finding is strongly supported by our ROV video footage (Videos [Link], [Link], [Link]), where we documented mesopelagic fish engaging in feeding frenzies on benthic prey.

### 3.2 Comparison Between the Prey Composition of *Benthosema* Taxa and *Vinciguerria mabahiss*

**FIGURE 2:**
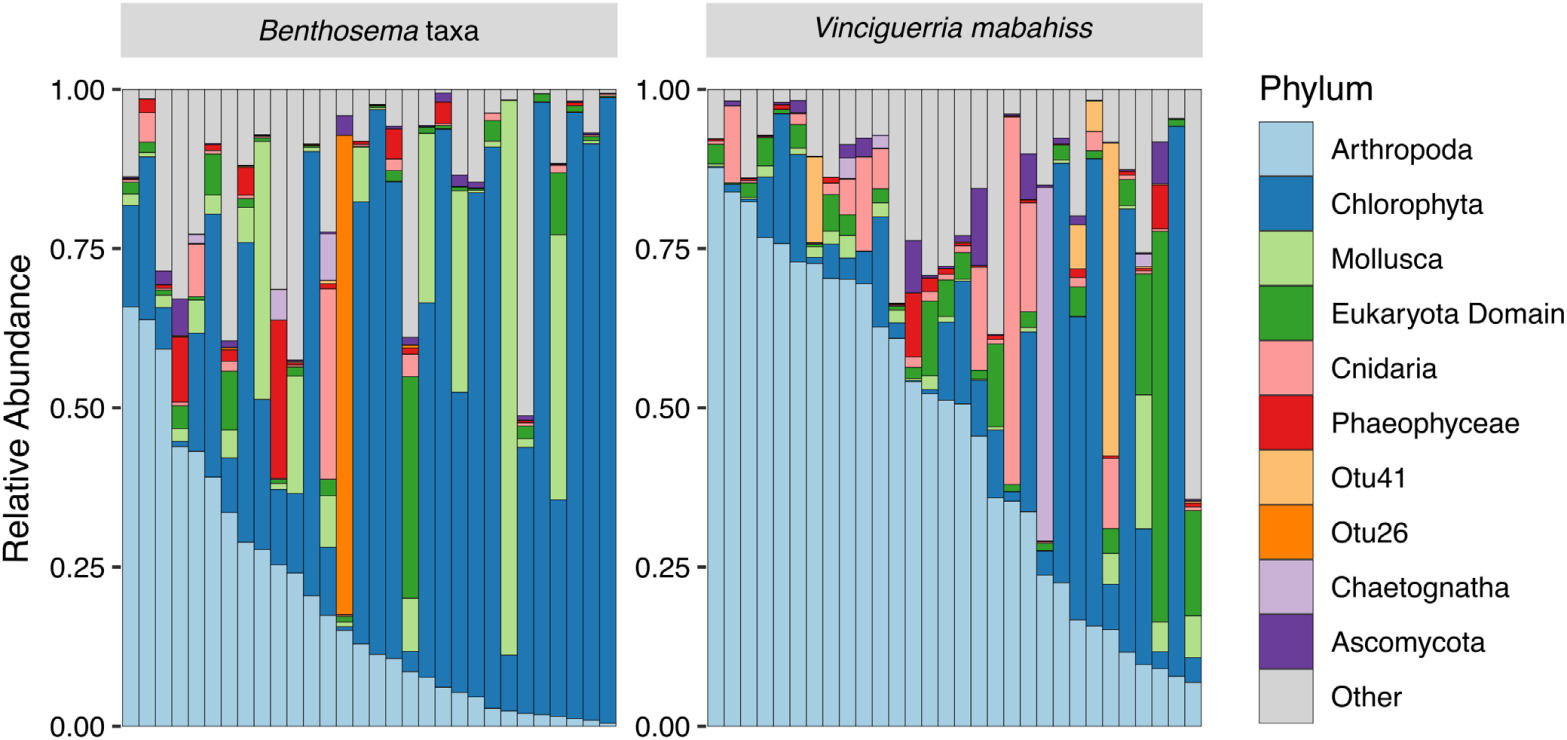
Prey items for each species of mesopelagic fish. Only the top 10 abundant phyla are illustrated. Each bar represents one individual, ordered according to the proportion of Arthropoda reads in their guts.

We observed a decreasing percentage in overlapping prey taxa between both host species as taxonomic resolution increased from phylum to species level (Figure S1 in Data S1). At the phylum level, the three most abundant prey taxa were Arthropoda, Chlorophyta, and Mollusca (Figure 2). Arthropods constituted more than 50% of the total prey reads in three *Benthosema* taxa and 16 *Vinciguerria mabahiss* samples, while chlorophytes represented over 50% of the total prey reads in 12 *Benthosema* taxa and four *V. mabahiss* samples. Additionally, molluscs contributed at least 25% of the total prey reads in five *Benthosema* taxa samples but only up to 20% to one *V. mabahiss* sample.

### 3.3 Prey Diversity and Composition

**FIGURE 3:**
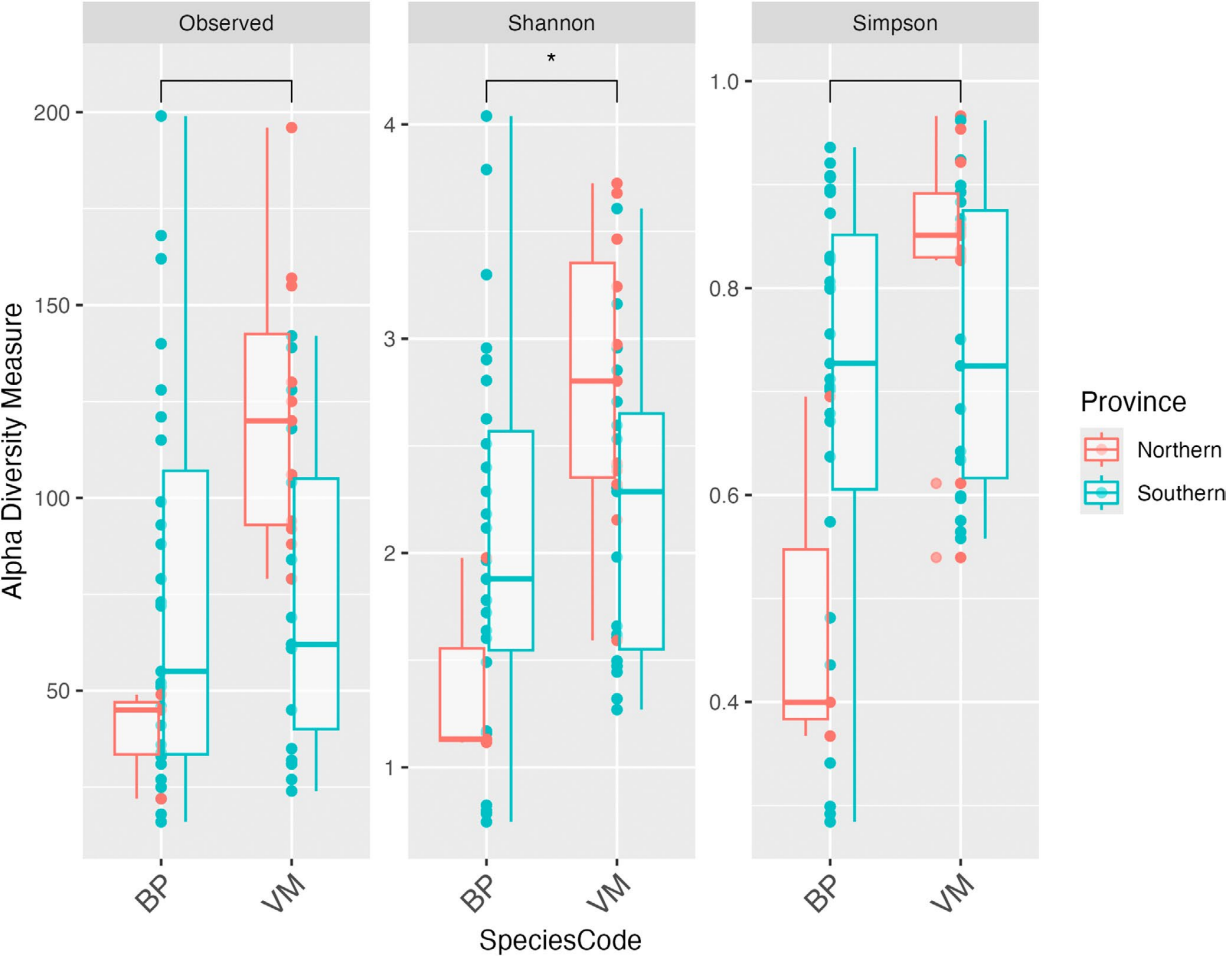
Comparison of alpha diversity estimates between the two mesopelagic fish species (BP—*Benthosema* taxa and VM— *Vinciguerria mabahiss* ) across two provinces in the Saudi Arabian Red Sea. From left to right, the graphs show observed OTU richness, the Shannon–Wiener diversity index, and the Simpson index. An asterisk (*) indicates a statistically significant (*p* < 0.05) difference in alpha diversity between the two species based on the Wilcoxon‐signed‐rank test.

To assess the diversity of the prey communities, all samples were rarefied to an even sequencing depth, leading to the exclusion of 21 OTUs from the analysis. *Vinciguerria mabahiss* exhibited higher alpha prey diversity indices compared to *Benthosema* taxa, regardless of province defined in the Red Sea (Figure 3). The median observed OTU richness of *V. mabahiss* was higher than that of *Benthosema* taxa, but the difference was not significant ($X^{2}$ = 0.5576, *p* > 0.05). The ecological diversity, measured by the Shannon‐Wiener diversity index, indicated significantly higher prey diversity in the gut samples of *V. mabahiss* compared to that of *Benthosema* taxa ($X^{2}$ = 4.346, *p* < 0.05). The Simpson diversity index was also higher in *V. mabahiss* compared to *Benthosema* taxa, but this difference was not significant ($X^{2}$ = 2.891, *p* > 0.05).

**FIGURE 4:**
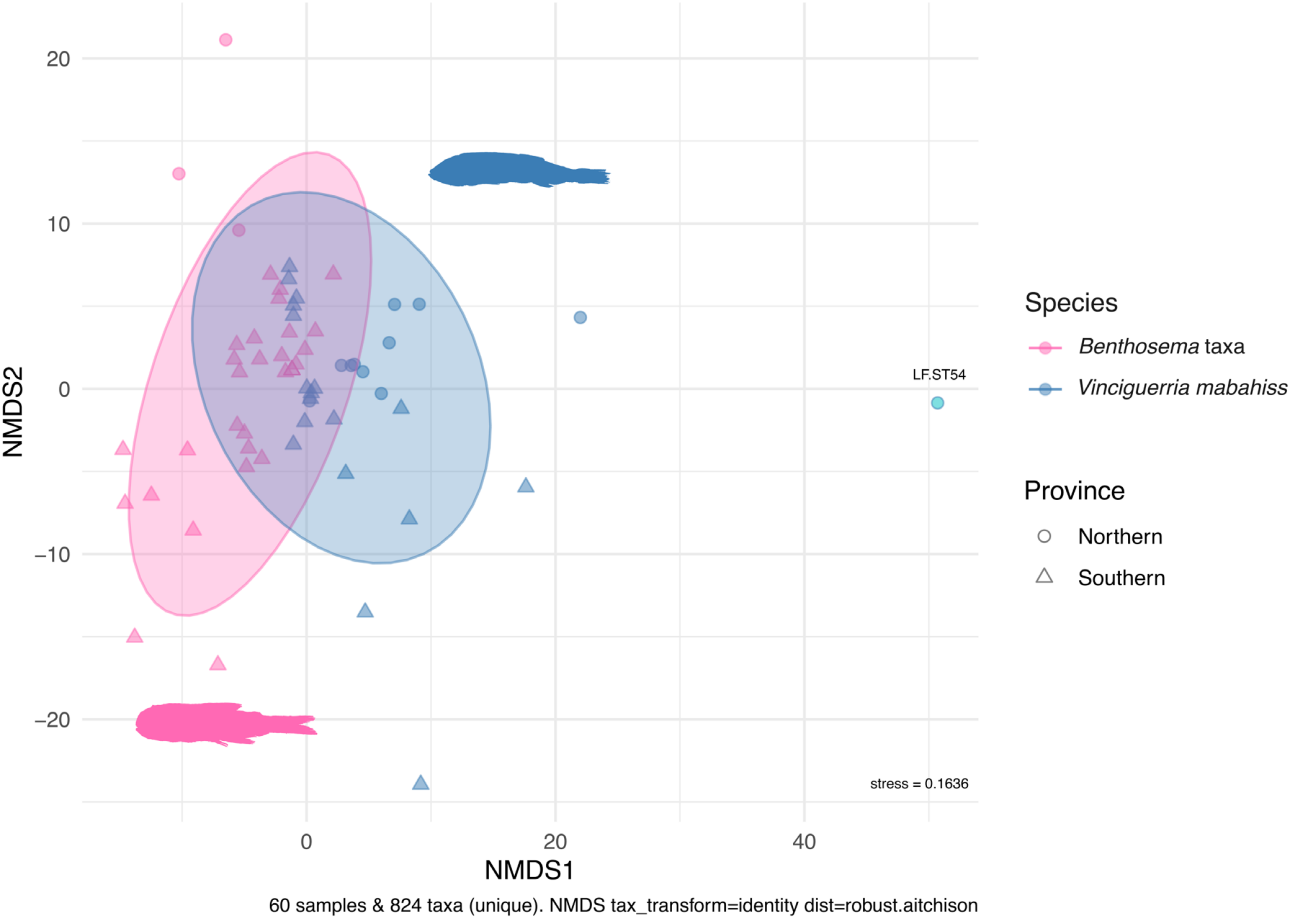
Non‐metric multidimensional scaling (NMDS) ordination of prey items used by the two mesopelagic fish species, based robust Aitchison distance. Ellipses represent the spread and overlap of the two species in two provinces, indicating the clustering and separation of the groups.

Non‐metric multidimensional scaling (NMDS) ordination based on the robust Aitchison distance revealed clustering of the two host species but no clear separation among the two defined provinces within the Red Sea (stress = 0.164, Figure 4). PERMANOVA analysis revealed that species (d*f* = 1, *F* = 3.543, *R* ^2^ = 0.055, *p* < 0.01) was a more significant factor than province (d*f* = 1, *F* = 2.534, *R* ^2^ = 0.040, *p* < 0.01) in explaining dietary differences. All groups showed homogeneous dispersion in the ordination space based on their centroids (PERMDISP_species_: d*f* = 1, *F* = 0.187, *p* = 0.67; PERMDISP_province_: d*f* = 1, *F* = 0.2677, *p* = 0.61).

### 3.4 Bipartite Network of Metazoan Prey

**FIGURE 5:**
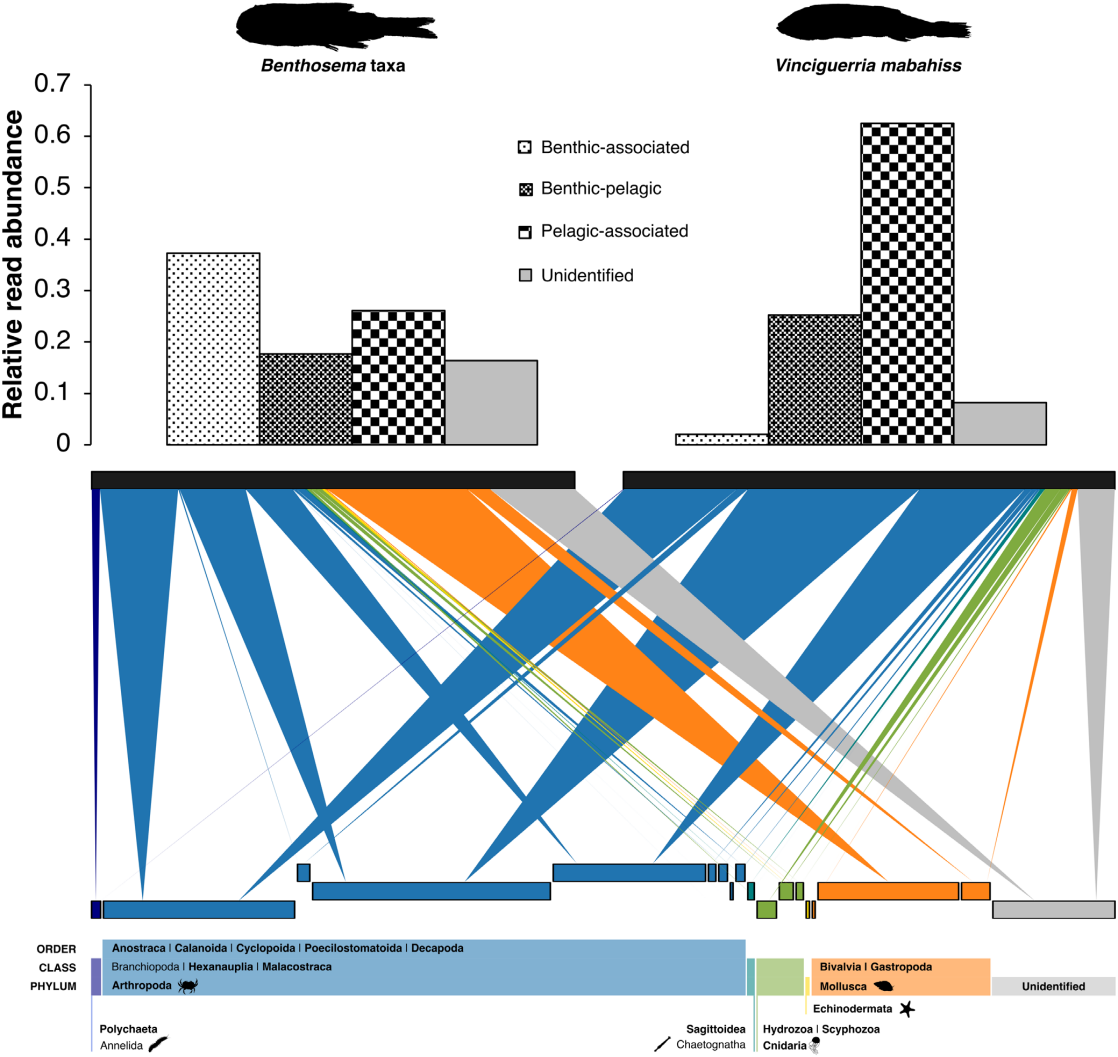
Bar charts (upper panel) displaying the distribution of the relative OTU read abundance of benthic, pelagic, benthic‐pelagic, and unidentified prey in each host species. The bipartite network (lower panel) illustrates the proportional use of prey items by the two host species. The thickness of the downward‐facing arrows represents the relative abundance of each prey taxon in the host species' gut contents. The height of the prey rectangles and labels reflects the level of taxonomic resolution from phylum (lowest) to class and order (highest). Bold letters mark taxa that have unique rectangles in the network. All labels and rectangles are ordered from left to right, corresponding to the colored bar position in the lower panel. Prey phyla are represented by distinct colors.

To focus on metazoan prey composition between the two species, non‐metazoan taxa (i.e., algae, fungi, and protists) were excluded from the bipartite network analysis. *Benthosema* taxa consisted of 18 times more benthic prey sequences and 0.4 times fewer pelagic prey sequences than *Vinciguerria mabahiss* , based on the relative OTU read abundance. The bipartite network of metazoan prey illustrated the relative read abundance for each taxon, indicated by the width of the downward arrows, across the two species at taxonomic resolution ranging from phylum to order level (Figure 5). Both species predominantly ingested arthropods, with varying degrees of feeding on molluscs and cnidarians at the phylum level. At the class level, *Benthosema* taxa had the highest proportional contribution of bivalves, while *V. mabahiss* had the highest proportion of copepods.

In terms of taxa‐specific prey comparison, *Benthosema* taxa had a higher proportion of polychaete sequence reads compared to *V. mabahiss* , while the latter had more cnidarian sequence reads. Echinoderm sequence reads were exclusive to *Benthosema* taxa, while both species contained chaetognath sequence reads attributed to the order Sagittoidea. At the species level, *Benthosema* taxa showed high selectivity towards unknown gastropod prey (Otu149), while *V. mabahiss* preferred unknown eukaryotes (Otu736) (Data S1). Four parasite phyla were detected in both species, with the highest reads recovered from apicomplexan parasites, followed by platyhelminths in *Benthosema* taxa and nematodes in *V. mabahiss* , along with a small proportion of nemerteans in both species (Figure S3 in Data S1). These findings highlight the distinct dietary strategies of each species and underscore the ecological significance of their prey selections.

### 3.5 Benthic Interaction From ROV Footage

Of the 17 video clips analyzed, nine (52.9%) documented mesopelagic fish interacting with the seafloor, displaying rapid darting movements indicative of potential benthic feeding behavior (see Videos [Link], [Link], [Link]). While precise species identification was challenging due to low video resolution and the swift movements of the fish, these interactions were frequently observed near the continental slope at the depth range of 496–778 m. Though the darting movements might initially appear to be startle responses to the ROV's presence and light, their consistent occurrence suggests natural feeding patterns. This observation aligns with the detection of benthic prey in gut content analyses, supporting the hypothesized benthic feeding behavior among mesopelagic fish.

## 4 Discussion

Our metabarcoding analysis confirms that both *Benthosema* taxa and *Vinciguerria mabahiss* predominantly feed on zooplankton, with calanoid copepods as the dominant prey. This finding supports the importance of zooplankton as the main food source in the Red Sea mesopelagic environment. Notably, *V. mabahiss* contained a 2.7‐fold higher relative abundance of arthropod sequence reads compared to *Benthosema* taxa, suggesting potential niche partitioning in their feeding strategies. While both species primarily consumed copepods, our study provides novel evidence of *Benthosema* taxa and *V. mabahiss* engaging in benthic feeding, an underreported behavior among mesopelagic fish.

### 4.1 Zooplankton as the Main Food Source

In the Red Sea, both species undergo normal diel vertical migration (NDVM) to the upper ~200 m of the water column at night, where calanoid copepods are abundant (Dypvik and Kaartvedt 2013; Klevjer et al. 2012). Although we did not directly measure prey abundance in their habitat, the notable prevalence of copepod reads in gut samples (class Hexanauplia, ~49% of metazoan prey) suggests efficient exploitation of available prey resources. This finding highlights the importance of these mesopelagic fish in the vertical transfer of biomass and nutrients within the Red Sea ecosystem.

Apart from arthropods, both fish species also consumed molluscs, cnidarians, and chaetognaths. The composition of the zooplankton community in the Red Sea is shaped by local oceanographic conditions, depth, topography, and seasonality (Casas et al. 2017; Pearman et al. 2014; Pearman and Irigoien 2015). Our sampling, conducted from February to June 2022 during the cold season (December to May), aligns with the dominance of these taxa in the mesopelagic fish diet, particularly arthropods and cnidarians, with smaller proportions of chaetognaths and molluscs (see Casas et al. 2017). It should be noted that the ingested zooplankton likely included larvae or juvenile stages, considering the relative sizes of predator and prey.

### 4.2 Novel Evidence of Benthic Feeding

Our study reveals a substantial presence of benthic prey sequences in the gut contents of *Benthosema* taxa, providing the first molecular evidence of mesopelagic fish in the Red Sea consuming benthic organisms, such as polychaetes, echinoderms, and gastropods. This challenges the traditional view of mesopelagic fish as strictly pelagic predators and suggests a more flexible, mixed diet that includes opportunistic benthic feeding. Video footage from ROV observations supports this finding, showing mesopelagic fish interacting with the continental shelf, with rapid darting movements suggestive of foraging behavior. Previous studies (Gartner et al. 2008; Klevjer et al. 2012) have hinted at similar behavior using acoustic and visual data but lacked direct confirmation from dietary analyses. Our study advances these observations with visual evidence of active benthic feeding (Videos [Link], [Link], [Link]), corroborated by gut content analyses revealing benthic taxa such as polychaetes, echinoderms, and gastropods.

### 4.3 Ecological and Physiological Considerations

Several hypotheses may explain the presence of benthic prey in mesopelagic fish diets: (1) opportunistic feeding on the seafloor during daytime descent; (2) attraction to the seafloor due to artificial light from the ROV; (3) consumption of benthic prey in larval form within the meroplankton community; (4) physiological adaptations that facilitate the exploitation of benthic resources near the slope; and (5) anatomical adaptations that enable the capture and processing of hard‐bodied prey.

The interactions observed between mesopelagic fish and the seafloor suggest that opportunistic feeding along the continental slope may occur as these fish descend to their daytime residence depths, typically between 500 and 750 m (Dypvik and Kaartvedt 2013). This descent coincides with periods of minimal pelagic feeding activity, raising the possibility that mesopelagic fish exploit benthic resources when they intersect continental slopes or underwater features during diel vertical migration (Gartner et al. 2008; Isaacs and Schwartz 1965; Mauchline and Gordon 1991). Supporting this hypothesis, we observed occasional associations of mesopelagic fish with benthic‐dwelling organisms, such as sea anemones, pandalid shrimps, and deep‐sea groupers (personal observation). Furthermore, the presence of benthic prey in their gut contents reinforces the hypothesis that mesopelagic fish incorporate benthic foraging into their feeding strategy. The attraction of Red Sea mesopelagic fish to artificial light is well documented (Dypvik and Kaartvedt 2013; Kaartvedt et al. 2019; Klevjer et al. 2012). As visual predators (Sabatés and Saiz 2000), mesopelagic fish may exhibit feeding responses to artificial light, influencing their interactions with benthic environments (Conley and Hopkins 2004).

Our findings suggest that the detected benthic prey items, particularly molluscs and echinoderms, likely represent larval forms within the meroplankton community. Notably, *Benthosema* taxa consumed a high number of molluscs, particularly bivalves, which constituted approximately one‐third of metazoan prey (Figure 5). This observation is consistent with a previous record of mollusk prey items (size between 0.5–1.5 mm) in the gut of *Benthosema pterotum* (Dalpadado and Gjosaeter 1988). The detection of echinoderms and polychaetes in the gut samples of *Benthosema* taxa—nearly absent in *Vinciguerria mabahiss* —suggests dietary specialization, possibly indicating opportunistic bottom feeding or selective targeting of larval forms.

Physiological adaptations may facilitate benthic feeding. Despite low oxygen conditions (< 1.5 mL O_2_ l^−1^) below 300 m in the Red Sea (Dypvik and Kaartvedt 2013), these fish have adapted to sustain high levels of activity, such as fast swimming and feeding. This adaptation is likely driven by the high metabolic demands associated with the relatively warm deep waters of the Red Sea (~21°C), necessitating frequent feeding to meet their energetic needs (see Klevjer et al. 2012 and references therein). Consequently, they may occasionally exploit benthic resources alongside their usual planktonic diet.

Additionally, anatomical adaptations play a crucial role in the feeding strategies of these fish. Studies suggest that predator agility, buccal part size, and dentition influence prey size and type (Alwis and Gjøsæter 1988; Legand et al. 1972; Martin and Davis 2016; Zavala‐Muñoz et al. 2019). The evolution of heterodonty in mesopelagic fish likely enhances their feeding ecology (Martin and Davis 2020). For instance, lanternfish such as *B. pterotum* possess specialized villiform teeth structures that enable them to secure hard‐bodied molluscs in the oral cavity. After securing the prey, they may utilize modified branchial teeth to crush hard shells, as observed in *Centrobranchus* spp. (Hopkins and Gartner 1992; Van Noord 2013; Watanabe et al. 2002). However, research on lightfish or related species is limited, hindering our understanding of prey preference based on dentition. Further exploration of tooth morphology in these fish is needed to inform their feeding ecology relative to depth stratification.

### 4.4 Species‐Specific Dietary Partitioning

Despite compositional similarities in prey communities, we observed significant differences in alpha diversity, with *Vinciguerria mabahiss* exhibiting a higher Shannon‐Wiener diversity index, indicating a more varied diet compared to *Benthosema* taxa. The beta diversity, measured by NMDS analysis, showed distinct clustering of prey OTUs by species, suggesting species‐specific dietary preferences with some overlap, likely due to shared prey resources. While copepods constituted the main diet of both host species, *Benthosema* taxa predominantly consumed hard‐bodied prey, including annelids, molluscs, and echinoderms, whereas *V. mabahiss* preferred gelatinous prey such as hydrozoans and scyphozoans. This dietary distinction likely minimizes direct competition between the species, facilitating their coexistence in the mesopelagic zone. These findings align with previous research documenting varied diet compositions and prey selectivity among mesopelagic fish species in the western Mediterranean Sea (Bernal et al. 2015). Furthermore, PERMANOVA results confirmed that species identity, rather than geographical region, is the primary factor influencing dietary differences, highlighting species‐specific ecological adaptations in feeding strategies.

### 4.5 The Role of Algae as Secondary Predation

Unexpectedly, we recovered a substantial read of algae in the gut contents of both fish species. Phytoplankton, particularly the class Chloropicophyceae, dominated the algae reads, comprising up to 65% of total prey reads. Chloropicophyceae, a recently described phytoplankton class (dos Lopes Santos et al. 2017), has not been previously reported in the Red Sea. Although the Red Sea is characterized as an oligotrophic environment with low nutrient availability, phytoplankton remain the foundation of the food web, contributing significantly to primary production (Al‐Otaibi et al. 2020; Coello‐Camba and Agustí 2021; Pearman et al. 2017). The presence of algae in fish gut contents likely reflects secondary predation. This occurs when dietary components of prey organisms are detected in the predator's gut—a common phenomenon observed in mesopelagic fish dietary studies employing metabarcoding (Clarke et al. 2020). The high abundance of algae in gut contents is unlikely to result from direct consumption, as the fish were captured during daylight hours at great depths, when feeding activity is minimal (Dypvik and Kaartvedt 2013). Instead, it reflects the dietary patterns of their primary prey, particularly copepods, which graze extensively on phytoplankton (Cornils et al. 2007; Sommer et al. 2002). Given the estimated digestion rate of mesopelagic fish (~12 h; Dypvik and Kaartvedt 2013), metabarcoding likely captured prey items at different stages of digestion, further supporting the secondary predation of algae. Further investigation is required to elucidate the exact mechanisms driving these dietary patterns.

### 4.6 Parasite Detection

The detection of parasites within fish guts underscores the potential role of mesopelagic fish as intermediate hosts in trophic interactions within deep‐sea ecosystems. These microscopic parasites are usually found in the organs (e.g., stomach, intestine, and mesenteries) and the body cavities of mesopelagic fish and therefore represent endoparasites. These endoparasites are not readily detectable by the naked eye, necessitating the application of genetic techniques. Myctophid‐specific parasites include nematodes (e.g., *Anisakis* spp., *Hysterothylacium aduncum*, *Phyllobothriidae*, *Elytrophalloides oatesi*) and digeneans ( *Gonocerca phycidis* and *Lethadena* sp.) (Cabrera‐Gil et al. 2018; Klimpel et al. 2010, 2008; Timi et al. 2024), which are common among the predators of myctophids (see Mateu et al. 2015 and references therein), suggesting that mesopelagic fish may serve as paratenic hosts in the life cycle of these parasites.

### 4.7 Ecological Implications

Our study provides new insights into the feeding ecology of mesopelagic fish in the Red Sea, particularly the role of benthic feeding. By integrating metabarcoding data and video evidence, we highlight the flexible foraging strategies employed by these fish, which may allow them to exploit both pelagic and benthic prey. This behavior has implications for nutrient transfer within deep‐sea ecosystems and the ecological roles of mesopelagic fish.

The discovery of benthic feeding necessitates a re‐evaluation of the role of mesopelagic fish in carbon sequestration. Given their substantial biomass in the Red Sea (Abu El‐Regal and Ditty 2023; Isari et al. 2017), these fish can contribute 10%–40% of deep ocean carbon export through defecation, respiration, extraction and predation during diel vertical migration (Saba et al. 2021). Recent estimates suggest that mesopelagic fish contribute approximately 16.1% (± 13%) to total carbon flux out of the euphotic zone (100–200 m), equivalent to 1.5 ± 1.2 Pg C per year (Saba et al. 2021). Their interactions with the benthic community suggest an additional pathway for nutrient transfer, warranting further exploration into how benthic feeding influences carbon cycling and the ecological impacts of these interactions.

While our findings emphasize the importance of benthic prey in *Benthosema* taxa, it is equally important to consider the role of gelatinous prey in the trophic dynamics of *Vinciguerria mabahiss* . Gelatinous zooplankton, often underrepresented in traditional gut content analyses due to rapid digestion (Arai et al. 2003; Purcell and Arai 2001), are crucial in deep‐sea food webs. The trophic significance of gelatinous prey has been observed in other regions, such as the Mid‐Atlantic Ridge, where gelativorous fish families contribute significantly to overall biomass (Sutton et al. 2008). Our metabarcoding results indicate that *V. mabahiss* shows a marked preference for gelatinous prey alongside copepods, suggesting that gelativory may also be an important trophic pathway in the Red Sea.

### 4.8 Limitations of the Study

The removal of host reads, which comprised 89.4% of total sequence reads, was crucial for obtaining accurate metabarcoding results, especially considering the potential for sample contamination from shared preservation in the same zip‐lock bags. As lanternfish are known to be piscivorous (Dalpadado and Gjosaeter 1988), host sequence reads may reflect cannibalism and trophic linkages between species rather than host materials, which cannot be resolved with the available data. Indeed, the small size of mesopelagic fish necessitated processing their entire alimentary tract, leading to a significant proportion of host‐derived sequences. Caution is therefore warranted when interpreting relative sequence abundances of prey items (Casey et al. 2019; Deagle et al. 2019). Moreover, the high proportion of unidentified OTUs underscores the need for comprehensive DNA barcode libraries in the region. While combining morphological and genetic analyses may improve taxonomic assignments, identifying prey items remains challenging due to the database gaps and morphological constraints.

## 5 Conclusion

Our study reveals distinct dietary patterns among mesopelagic fish in the Red Sea, particularly *Benthosema* taxa and *Vinciguerria mabahiss* . Both species consume a mix of planktonic and benthic prey, with arthropods dominating their diets. However, their dietary divergence—characterized by *Benthosema* taxa's preference for benthic prey and *V. mabahiss* 's reliance on gelatinous plankton—demonstrates niche partitioning that likely minimizes interspecific competition in this nutrient‐poor ecosystem. The rare observation of mesopelagic fish interacting with the benthic community in ROV footage highlights a previously overlooked aspect of their feeding ecology. While our gut content analysis did not directly target the individuals observed engaging in benthic feeding, these findings suggest that benthic prey may play a more significant role in mesopelagic fish diets than previously recognized. Understanding these diet dynamics is essential for accurately assessing the biogeochemical roles of these fish, particularly in carbon sequestration through their daily vertical migrations. Future research integrating DNA metabarcoding with stable isotope analysis will be essential to further refine our understanding of diet partitioning and the ecological contributions of mesopelagic fish in this ecosystem. These insights are vital for informing conservation and management strategies aimed at preserving these ecologically and economically important species and their habitat.

## Author Contributions

**Kah Kheng Lim:** conceptualization (equal), data curation (lead), formal analysis (lead), methodology (lead), resources (equal), software (equal), visualization (lead), writing – original draft (lead). **Carlos Angulo‐Preckler:** conceptualization (equal), methodology (equal), project administration (supporting), resources (equal), writing – review and editing (equal). **Christopher A. Hempel:** conceptualization (supporting), data curation (supporting), methodology (supporting), software (equal), writing – review and editing (equal). **Mohammad A. Qurban:** funding acquisition (equal), resources (equal), resources (equal), writing – review and editing (equal), writing – review and editing (equal). **Vincent A. Pieribone:** resources (equal). **Carlos M. Duarte:** conceptualization (equal), funding acquisition (equal), supervision (lead), writing – review and editing (equal).

## Conflicts of Interest

The authors declare no conflicts of interest.

## Supporting information


**Data S1.** Supporting Information.

**Video S1.** Supporting Information.

**Video S2.** Supporting Information.

**Video S3.** Supporting Information.

## Data Availability

The raw sequencing data used in this study is available in NCBI with the BioProject ID: PRJNA1179974 and R script can be found in the publicly accessible repository (https://github.com/limkahkheng/mesopelagic‐fish‐gut‐content‐analysis.git).
