# Supplementary material for: Benthic Feeding and Diet Partitioning in Red Sea Mesopelagic Fish Resolved Through DNA Metabarcoding and ROV Footage
Source: Ecol Evol. 2025 Mar 6;15(3):e71091. doi: 10.1002/ece3.71091 (PMC11884935; doi:10.1002/ece3.71091)
Supplement: Supplementary file 1 — Data S1. Supporting Information. [file ECE3-15-e71091-s001.docx]

**Supplemental Information for:**

**Benthic feeding and diet partitioning in Red Sea mesopelagic fish resolved through DNA metabarcoding and ROV footage**

Kah Kheng Lim^*1^, Carlos Angulo-Preckler^1^, Christopher A. Hempel^1^, Mohammad A. Qurban^2^, Vincent A. Pieribone^3^, Carlos M. Duarte^1^

^1^Marine Science Program, Biological and Environmental Science and Engineering Division, King Abdullah University of Science and Technology (KAUST), Thuwal, 23955-6900, Kingdom of Saudi Arabia

^2^ National Center for Wildlife (NCW), PP2R+WJH, Makkah Al Mukarramah Branch Rd, King Abdul Aziz, Riyadh 12411, Kingdom of Saudi Arabia

^3^ OceanX, 37 West 39th St, 8th Floor, New York, NY 10018, U.S.A.

^*^Corresponding author: [kahkheng.lim@kaust.edu.sa](mailto:kahkheng.lim@kaust.edu.sa)

**Supplementary Table 1**. Distribution of mesopelagic fish samples by dive number, species, standard length (SL), and geographic location in the Red Sea.

| **Dive no.** | **Bp** | **SL (mm)** | **Vm** | **SL (mm)** | **Latitude** | **Longitude** | **Province** |
| --- | --- | --- | --- | --- | --- | --- | --- |
| CHR0188 | 1 | 42.53 | 0 | - | 20.180538 | 39.306439 | Southern |
| CHR0191 | 2 | 31.36–34.36 | 0 | - | 19.366245 | 39.276706 | Southern |
| CHR0199 | 0 | - | 7 | 18.09–27.75 | 21.425488 | 39.030606 | Southern |
| CHR0201 | 2 | 36.94–39.04 | 2 | 22.69–28.29 | 20.635875 | 39.467614 | Southern |
| CHR0202 | 0 | - | 4 | 20.8–28.4 | 20.24747 | 39.508776 | Southern |
| CHR0203 | 0 | - | 1 | 24.78 | 19.856452 | 40.126386 | Southern |
| CHR0211 | 2 | 22.3–26.22 | 0 | - | 18.521974 | 40.196720 | Southern |
| CHR0236 | 10 | 25.29–33.04 | 0 | - | 18.265658 | 40.973845 | Southern |
| CHR0240 | 10 | 31.9–36.94 | 0 | - | 18.990820 | 40.616719 | Southern |
| CHR0254 | 0 | - | 3 | 24.6127.25 | 22.413486 | 38.676353 | Southern |
| CHR0259 | 0 | - | 4 | 22.92–24.41 | 24.591034 | 36.920500 | Northern |
| CHR0265 | 0 | - | 2 | 18.19–21.16 | 22.484302 | 38.865877 | Southern |
| CHR0269 | 0 | - | 2 | 19.84–20.65 | 23.798929 | 38.202857 | Northern |
| CHR0275 | 0 | - | 1 | 24.71 | 25.768232 | 36.514866 | Northern |
| CHR0281 | 0 | - | 3 | 24.26–25.17 | 25.794715 | 36.363139 | Northern |
| CHR0285 | 1 | 44.69 | 0 | - | 27.090971 | 35.645153 | Northern |
| CHR0287 | 1 | n/a | 0 | - | 29.169718 | 34.866607 | Northern |
| CHR0291 | 1 | 24.44 | 0 | n/a | 28.350258 | 34.697070 | Northern |
| CHR0294 | 0 | - | 1 | - | 26.921353 | 35.741576 | Northern |

Note: The table includes the number of individuals of Benthosema taxa (Bp) and Vinciguerria mabahiss (Vm) used for gut content analysis, their standard lengths (SL, in mm), and the corresponding dive latitude, longitude, and province.

**Supplementary Table 2.** Operational Taxonomic Unit (OTU) table generated from the bioinformatic pipeline used for this study. This table includes the cleaned dataset, with contaminants, host sequences, and terrestrial sequences removed. These removed sequences are retained in a separate tab within the same Excel file for reference.


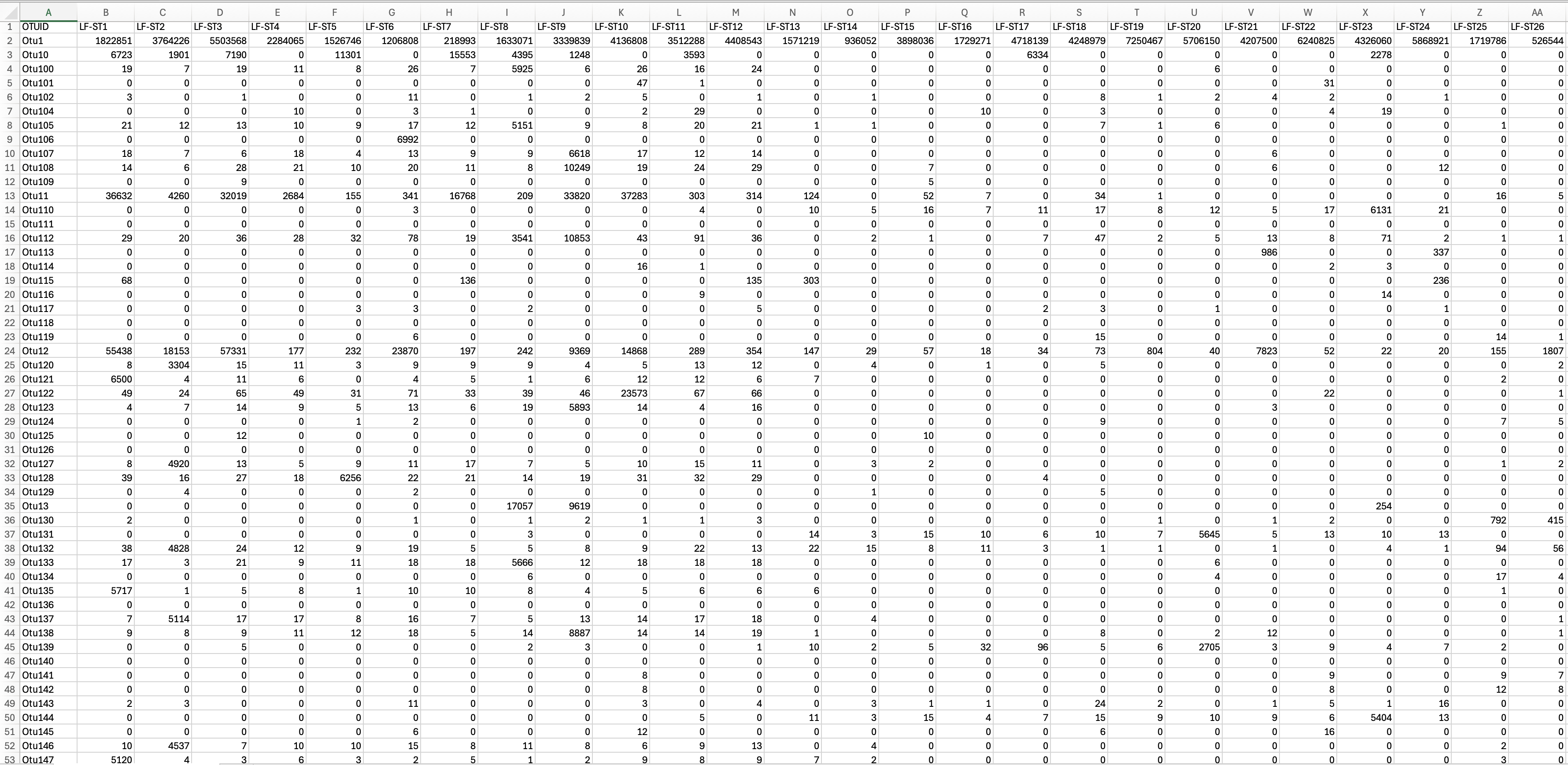


<https://figshare.com/s/68d7cb548fbde4fbe0b7>

**Supplementary Figure 1**. Venn diagrams showing the number of prey taxa recovered identified in the gut contents of Benthosema taxa (pink) and Vinciguerria mabahiss (blue) at different taxonomic ranks. The blow-up diagram at the top shows the prey taxa recovered from both fish species at the Order level.


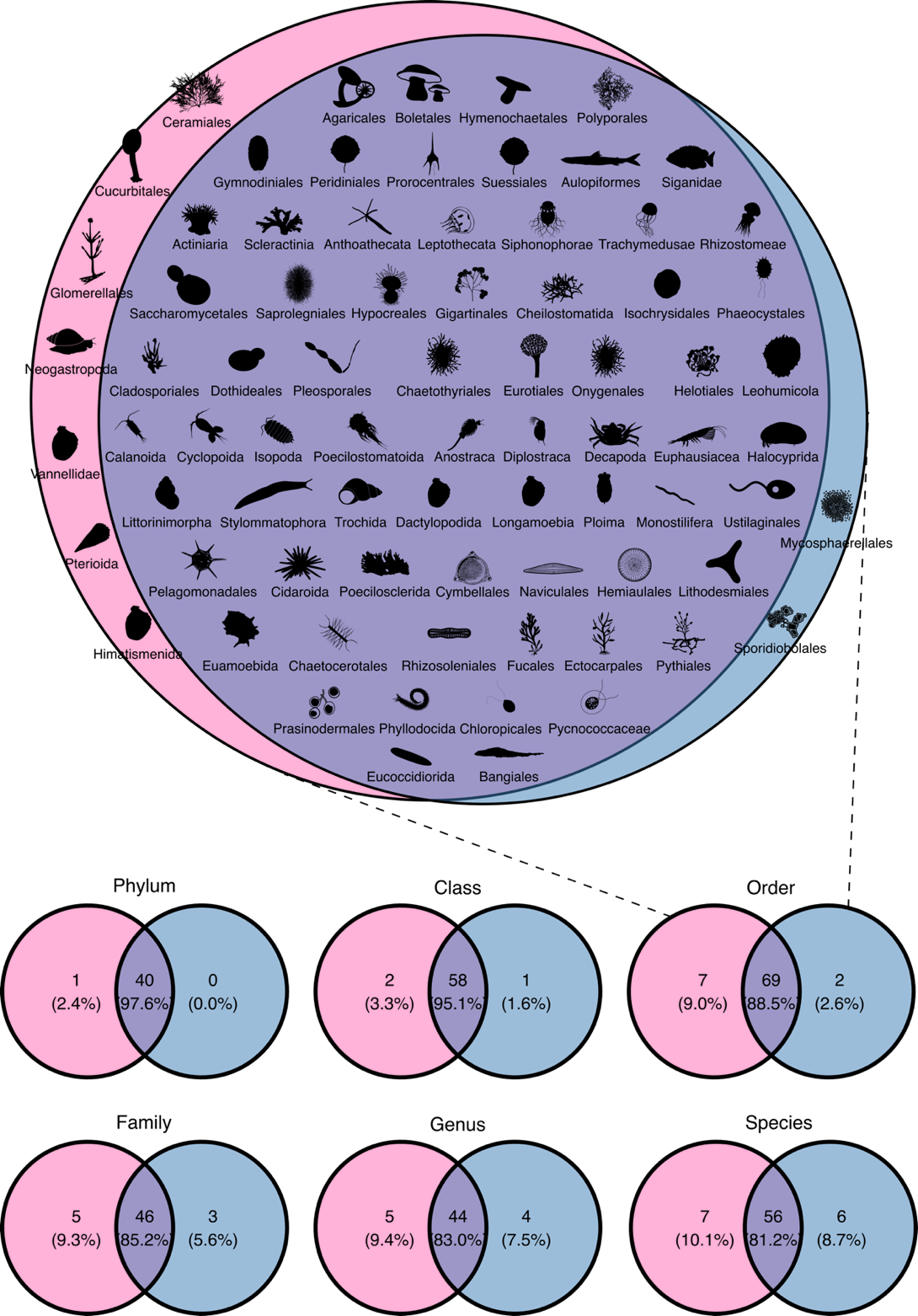


**Supplementary Figure 2**. Indicator species of prey taxa for mesopelagic fish in the Red Sea. Only the top ten significant taxa are displayed.


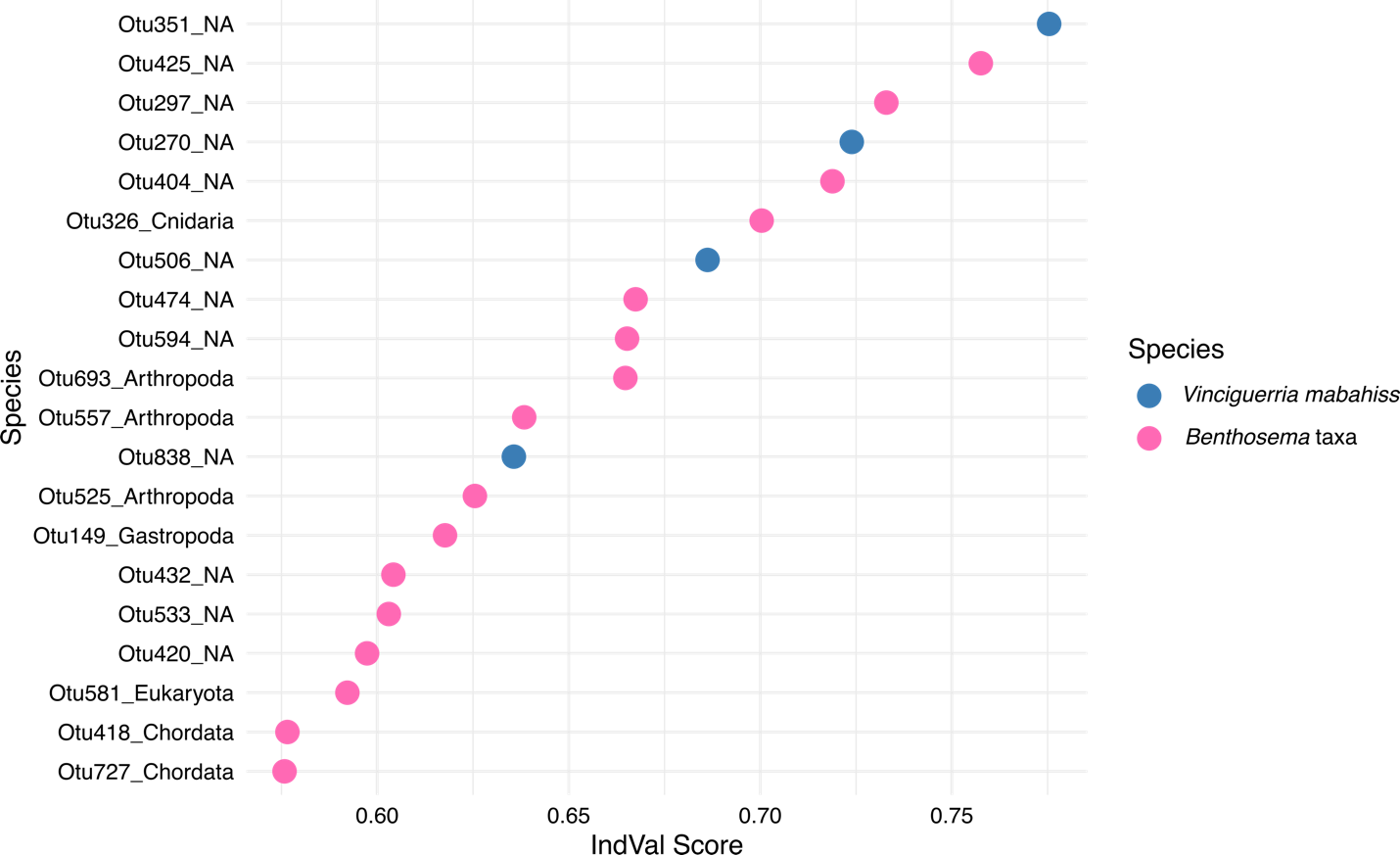


**Supplementary Figure 3**. Parasite phyla detected in both mesopelagic fish species.


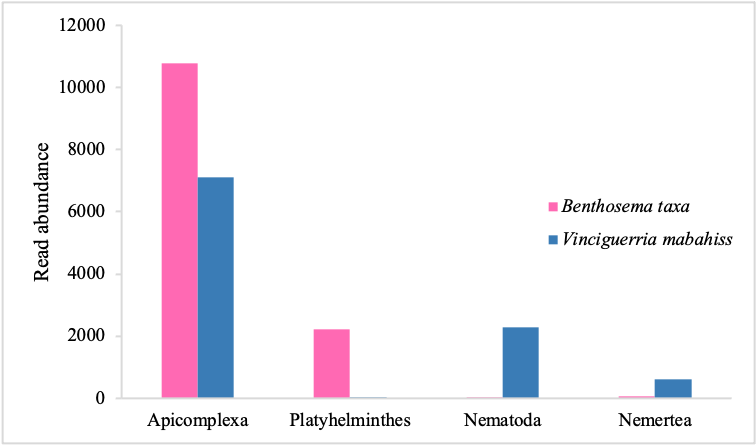


**Supplementary Video 1-3**. Videos showing mesopelagic fish darting near the seafloor, potentially feeding on prey in the muddy substrate (possibly ooze). The sediment clouds illustrate the disturbance to the seafloor caused by their rapid darting movements. Although not analyzed in this study, the ROV footage from Dive CHR0223 (Supplementary Video 3) provides the clearest view of this benthic feeding behaviour.


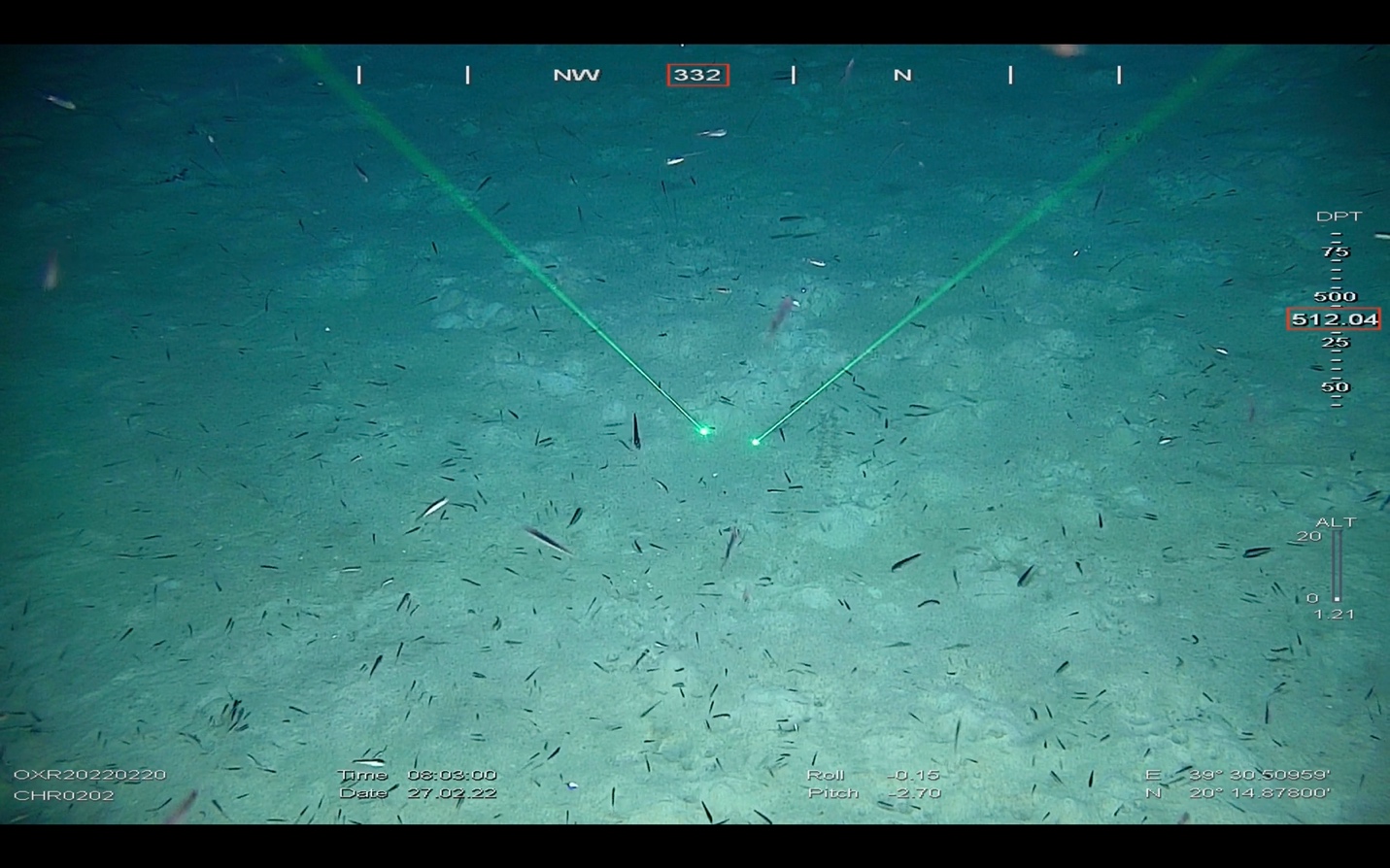


<https://figshare.com/s/527d92ccfae481bbdb4f>


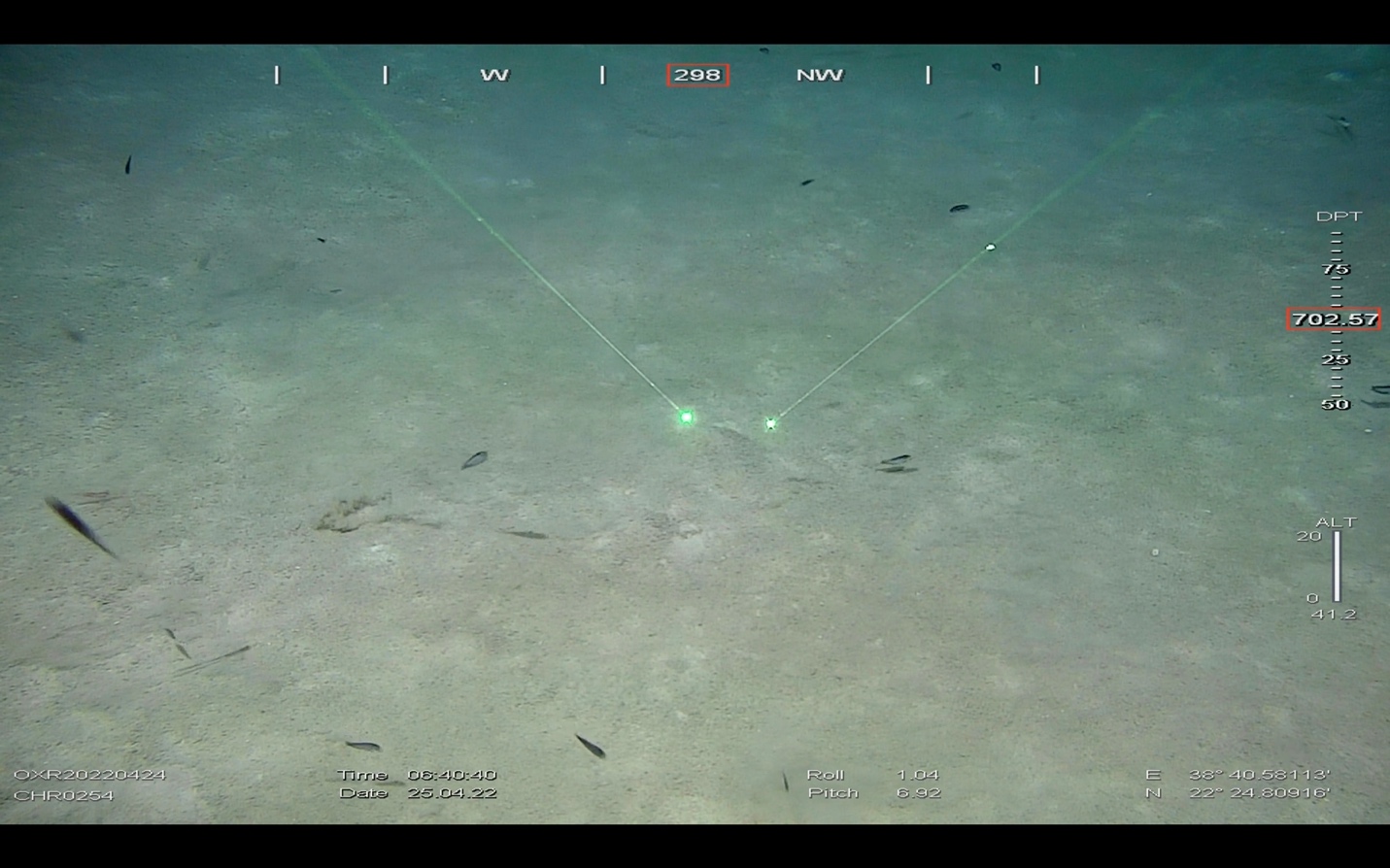


<https://figshare.com/s/546713991c0c58faba96>


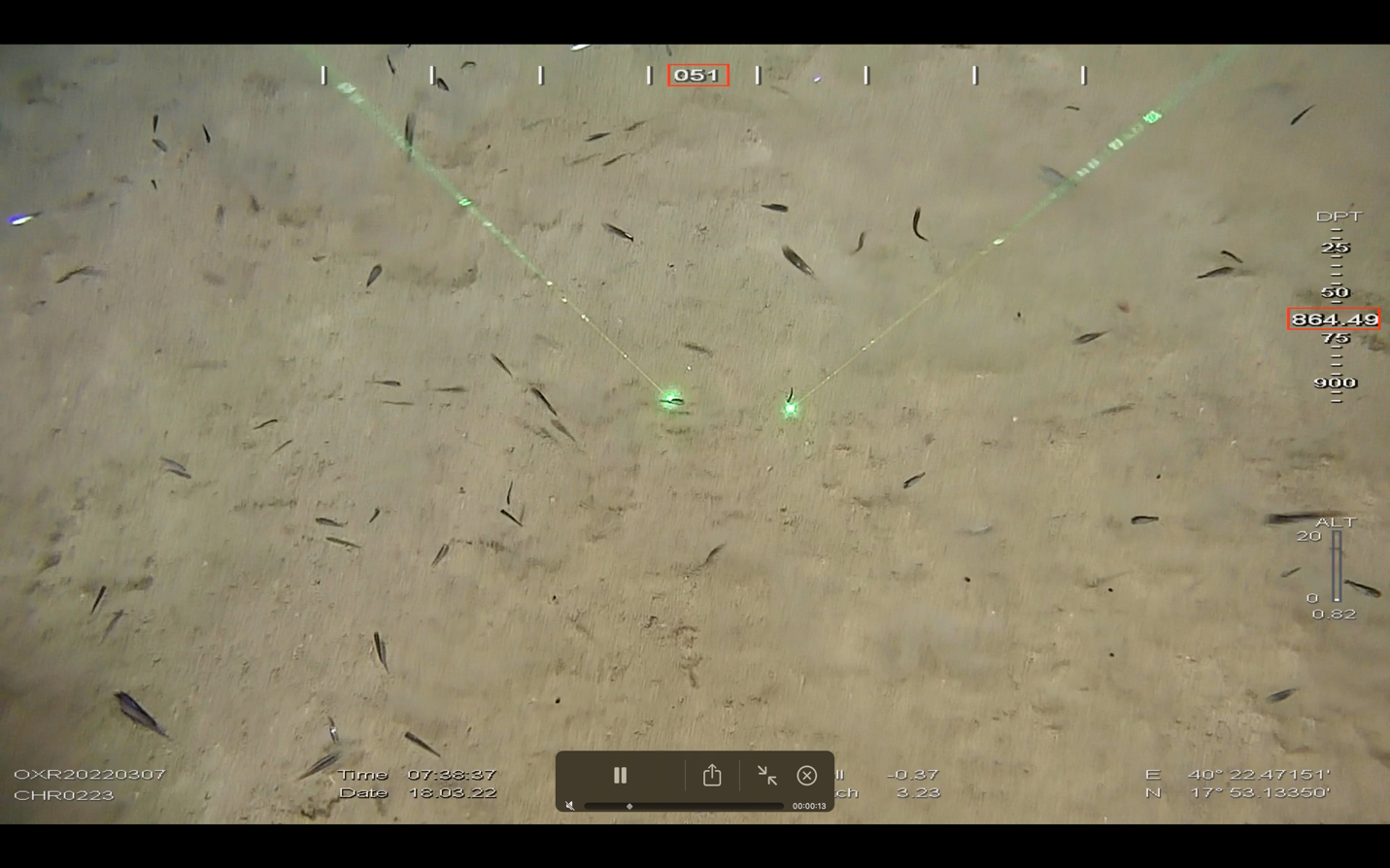


<https://figshare.com/s/495f402fa5c50fa00e05>
